# 4-Chloro-*N*-*o*-tolyl­benzamide

**DOI:** 10.1107/S1600536808030912

**Published:** 2008-09-30

**Authors:** Aamer Saeed, Rasheed Ahmad Khera, Naeem Abbas, Kazuma Gotoh, Hiroyuki Ishida

**Affiliations:** a Department of Chemistry, Quaid-I-Azam University, Islamabad 45320, Pakistan; b Department of Chemistry, Faculty of Science, Okayama University, Okayama 700-8530, Japan

## Abstract

In the mol­ecule of the title compound, C_14_H_12_ClNO, the two benzene rings are close to coplanar [dihedral angle = 7.85 (4)°]. The amide N—C=O plane makes dihedral angles of 34.04 (4) and 39.90 (3)°, respectively, with the 4-chloro- and 2-methyl­phenyl rings. In the crystal structure, inter­molecular N—H⋯O hydrogen bonds link the mol­ecules into chains.

## Related literature

For a related structure, see: Saeed *et al.* (2008 ▶). For bond-length data, see: Allen *et al.* (1987 ▶).
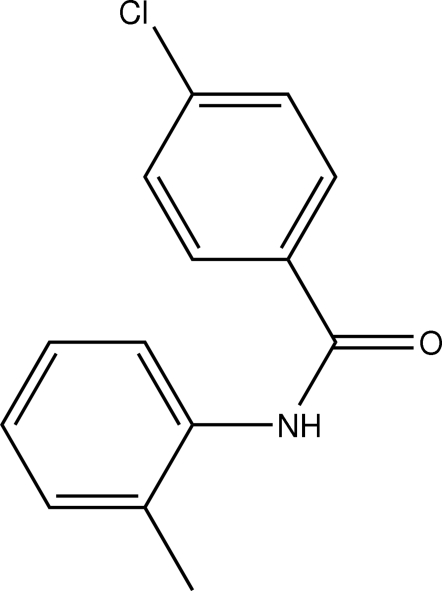


## Experimental

### 

#### Crystal data

C_14_H_12_ClNO
*M* *_r_* = 245.71
Monoclinic, 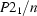

*a* = 10.7906 (14) Å
*b* = 4.8793 (6) Å
*c* = 23.522 (3) Å
β = 98.125 (3)°
*V* = 1226.0 (3) Å^3^
*Z* = 4
Mo *K*α radiation
μ = 0.29 mm^−1^
*T* = 223 (1) K
0.40 × 0.18 × 0.09 mm

#### Data collection

Rigaku R-AXIS RAPID II diffractometer
Absorption correction: numerical (*ABSCOR*; Higashi, 1999 ▶) *T*_min_ = 0.928, *T*_max_ = 0.974
14142 measured reflections
3461 independent reflections
1685 reflections with *I* > 2σ(*I*)
*R*_int_ = 0.046

#### Refinement

*R*[*F* ^2^ > 2σ(*F* ^2^)] = 0.049
*wR*(*F* ^2^) = 0.166
*S* = 1.06
3461 reflections
159 parameters
H atoms treated by a mixture of independent and constrained refinement
Δρ_max_ = 0.22 e Å^−3^
Δρ_min_ = −0.28 e Å^−3^

### 

Data collection: *PROCESS-AUTO* (Rigaku/MSC, 2004 ▶); cell refinement: *PROCESS-AUTO*; data reduction: *CrystalStructure* (Rigaku/MSC, 2004 ▶); program(s) used to solve structure: *SHELXS97* (Sheldrick, 2008 ▶); program(s) used to refine structure: *SHELXL97* (Sheldrick, 2008 ▶); molecular graphics: *ORTEP-3* (Farrugia, 1997 ▶) and *PLATON* (Spek, 2003 ▶); software used to prepare material for publication: *CrystalStructure* and *PLATON*.

## Supplementary Material

Crystal structure: contains datablocks global, I. DOI: 10.1107/S1600536808030912/hk2536sup1.cif

Structure factors: contains datablocks I. DOI: 10.1107/S1600536808030912/hk2536Isup2.hkl

Additional supplementary materials:  crystallographic information; 3D view; checkCIF report

## Figures and Tables

**Table 1:** Hydrogen-bond geometry (Å, °)

*D*—H⋯*A*	*D*—H	H⋯*A*	*D*⋯*A*	*D*—H⋯*A*
N1—H1⋯O1^i^	0.86 (2)	2.07 (2)	2.9073 (18)	164.1 (17)

Symmetry code: (i) 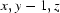.

**Fig. 1.**
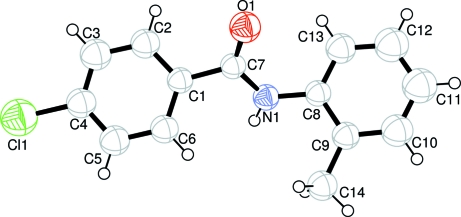
The molecular structure of the title molecule, with the atom-numbering scheme.

**Fig. 2.**
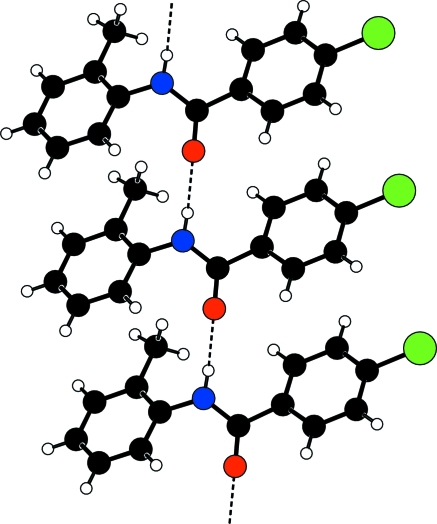
A partial packing diagram of the title compound. Hydrogen bonds are shown as dashed lines.

## supplementary crystallographic information

### Comment

The background to this study has been described in our earlier paper of 4-chloro-*N*-(2-chlorophenyl)-benzamide (Saeed *et al.*, 2008).

In the molecule of the title compound (Fig. 1), the bond lengths (Allen *et al.*, 1987) and angles are within normal ranges. Rings A (C1–C6) and B (C8–C13) are, of course, planar and the dihedral angle between them is 7.85 (4)°. So, they are also nearly coplanar. The amide plane (N1/O1/C7) is oriented with respect to rings A and B at dihedral angles of 34.04 (4)° and 39.90 (3)°, respectively.

In the crystal structure, intermolecular N—H···O hydrogen bonds (Table 1) link the molecules into chains (Fig. 2), in which they may be effective in the stabilization of the structure.

### Experimental

For the preparation of the title compound, 4-chorobenzoyl chloride (5.4 mmol) in CHCl_3_ was treated with 2-methylaniline (21.6 mmol) under a nitrogen atmosphere at reflux for 4 h. Upon cooling, the reaction mixture was diluted with CHCl_3_ and washed consecutively with aq. 1 *M* HCl and saturated aq. NaHCO_3_. The organic layer was dried over anhydrous sodium sulfate and concentrated under reduced pressure. Crystallization of the residue in CHCl_3_ afforded the title compound (yield; 84%). Anal. calcd. for C_14_H_12_ClNO: C 58.67, H 4.92, N 5.70%; found: C 58.61, H 4.96, N 5.79%.

### Refinement

The methyl H atoms were disordered. During the refinement process the disordered atoms were refined with occupancies of 0.80 (2) and 0.20 (2). H1 atom (for NH) was located in difference synthesis and refined isotropically [N—H = 0.86 (2) Å and U_iso_(H) = 0.067 (6) Å^2^]. The remaining H atoms were positioned geometrically, with C—H = 0.94 and 0.97 Å for aromatic and methyl H, respectively, and constrained to ride on their parent atoms with U_iso_(H) = xU_eq_(C), where x = 1.5 for methyl H and x = 1.2 for aromatic H atoms.

### Crystal data

**Table:**

C_14_H_12_ClNO	*F*(000) = 512.00
*M**_r_* = 245.71	*D*_x_ = 1.331 Mg m^−^^3^
Monoclinic, *P*2_1_/*n*	Mo *K*α radiation, λ = 0.71075 Å
Hall symbol: -P 2yn	Cell parameters from 7779 reflections
*a* = 10.7906 (14) Å	θ = 3.0–30.0°
*b* = 4.8793 (6) Å	µ = 0.29 mm^−^^1^
*c* = 23.522 (3) Å	*T* = 223 K
β = 98.125 (3)°	Block, colorless
*V* = 1226.0 (3) Å^3^	0.40 × 0.18 × 0.09 mm
*Z* = 4	

### Data collection

**Table:**

Rigaku R-AXIS RAPID II diffractometer	1685 reflections with *I* > 2σ(*I*)
Detector resolution: 10.00 pixels mm^-1^	*R*_int_ = 0.046
ω scans	θ_max_ = 30.0°, θ_min_ = 3.0°
Absorption correction: numerical (ABSCOR; Higashi, 1999)	*h* = −15→15
*T*_min_ = 0.928, *T*_max_ = 0.974	*k* = −6→6
14142 measured reflections	*l* = −33→33
3461 independent reflections	

### Refinement

**Table:**

Refinement on *F*^2^	H atoms treated by a mixture of independent and constrained refinement
*R*[*F*^2^ > 2σ(*F*^2^)] = 0.049	*w* = 1/[σ^2^(*F*_o_^2^) + (0.0782*P*)^2^] where *P* = (*F*_o_^2^ + 2*F*_c_^2^)/3
*wR*(*F*^2^) = 0.166	(Δ/σ)_max_ < 0.001
*S* = 1.06	Δρ_max_ = 0.22 e Å^−^^3^
3461 reflections	Δρ_min_ = −0.28 e Å^−^^3^
159 parameters	

### Special details

**Table:**

Geometry. All e.s.d.'s (except the e.s.d. in the dihedral angle between two l.s. planes) are estimated using the full covariance matrix. The cell e.s.d.'s are taken into account individually in the estimation of e.s.d.'s in distances, angles and torsion angles; correlations between e.s.d.'s in cell parameters are only used when they are defined by crystal symmetry. An approximate (isotropic) treatment of cell e.s.d.'s is used for estimating e.s.d.'s involving l.s. planes.
Refinement. Refinement of *F*^2^ against ALL reflections. The weighted *R*-factor *wR* and goodness of fit *S* are based on *F*^2^, conventional *R*-factors *R* are based on *F*, with *F* set to zero for negative *F*^2^. The threshold expression of *F*^2^ > σ(*F*^2^) is used only for calculating *R*-factors(gt) *etc*. and is not relevant to the choice of reflections for refinement. *R*-factors based on *F*^2^ are statistically about twice as large as those based on *F*, and *R*- factors based on ALL data will be even larger.

### Fractional atomic coordinates and isotropic or equivalent isotropic displacement parameters (Å^2^)

**Table:**

	*x*	*y*	*z*	*U*_iso_*/*U*_eq_	Occ. (<1)
Cl1	0.40808 (7)	0.32489 (17)	0.27042 (3)	0.1042 (3)	
O1	0.31241 (13)	0.9179 (2)	0.51539 (6)	0.0709 (4)	
N1	0.25683 (14)	0.4881 (3)	0.53727 (6)	0.0523 (4)	
H1	0.2591 (18)	0.319 (4)	0.5268 (8)	0.067 (6)*	
C1	0.32795 (16)	0.5743 (3)	0.44582 (8)	0.0509 (4)	
C2	0.42185 (17)	0.7014 (4)	0.42126 (10)	0.0653 (5)	
H2	0.4689	0.8416	0.4414	0.078*	
C3	0.4477 (2)	0.6259 (4)	0.36758 (10)	0.0750 (6)	
H3	0.5129	0.7107	0.3515	0.090*	
C4	0.3762 (2)	0.4243 (4)	0.33802 (9)	0.0676 (5)	
C5	0.2816 (2)	0.2953 (4)	0.36098 (9)	0.0686 (5)	
H5	0.2336	0.1583	0.3402	0.082*	
C6	0.25799 (18)	0.3701 (4)	0.41517 (8)	0.0600 (5)	
H6	0.1940	0.2817	0.4314	0.072*	
C7	0.29972 (15)	0.6742 (3)	0.50259 (8)	0.0496 (4)	
C8	0.21114 (17)	0.5490 (3)	0.58983 (7)	0.0520 (4)	
C9	0.10277 (18)	0.4181 (4)	0.60191 (8)	0.0579 (5)	
C10	0.0587 (2)	0.4874 (5)	0.65275 (9)	0.0779 (6)	
H10	−0.0138	0.4006	0.6618	0.093*	
C11	0.1170 (3)	0.6773 (6)	0.68999 (10)	0.0923 (8)	
H11	0.0837	0.7228	0.7236	0.111*	
C12	0.2238 (3)	0.8005 (5)	0.67811 (10)	0.0905 (8)	
H12	0.2645	0.9300	0.7038	0.109*	
C13	0.2728 (2)	0.7363 (4)	0.62834 (9)	0.0700 (6)	
H13	0.3473	0.8191	0.6207	0.084*	
C14	0.03458 (18)	0.2127 (4)	0.56166 (10)	0.0674 (5)	
H14A	0.0089	0.2980	0.5246	0.101*	0.80 (2)
H14B	−0.0387	0.1481	0.5772	0.101*	0.80 (2)
H14C	0.0894	0.0592	0.5571	0.101*	0.80 (2)
H14D	0.0782	0.1894	0.5287	0.101*	0.20 (2)
H14E	−0.0498	0.2772	0.5490	0.101*	0.20 (2)
H14F	0.0312	0.0386	0.5813	0.101*	0.20 (2)

### Atomic displacement parameters (Å^2^)

**Table:**

	*U*^11^	*U*^22^	*U*^33^	*U*^12^	*U*^13^	*U*^23^
Cl1	0.1230 (6)	0.1310 (7)	0.0666 (4)	0.0156 (4)	0.0405 (4)	−0.0055 (3)
O1	0.0955 (10)	0.0364 (6)	0.0858 (10)	−0.0028 (6)	0.0300 (8)	−0.0084 (6)
N1	0.0646 (9)	0.0387 (7)	0.0550 (9)	0.0004 (7)	0.0135 (7)	−0.0055 (6)
C1	0.0528 (9)	0.0422 (8)	0.0589 (10)	0.0044 (7)	0.0127 (8)	0.0008 (7)
C2	0.0615 (11)	0.0564 (11)	0.0825 (14)	−0.0036 (9)	0.0262 (10)	−0.0072 (10)
C3	0.0733 (13)	0.0705 (13)	0.0893 (16)	0.0016 (11)	0.0393 (12)	0.0029 (12)
C4	0.0751 (12)	0.0740 (13)	0.0573 (11)	0.0188 (11)	0.0219 (10)	0.0049 (10)
C5	0.0737 (12)	0.0730 (13)	0.0589 (12)	−0.0012 (11)	0.0084 (10)	−0.0065 (10)
C6	0.0647 (11)	0.0613 (11)	0.0558 (11)	−0.0047 (9)	0.0148 (8)	−0.0007 (8)
C7	0.0511 (9)	0.0370 (8)	0.0618 (11)	0.0035 (7)	0.0114 (8)	−0.0008 (7)
C8	0.0647 (10)	0.0437 (9)	0.0469 (9)	0.0117 (8)	0.0061 (8)	−0.0010 (7)
C9	0.0674 (11)	0.0560 (10)	0.0516 (10)	0.0160 (9)	0.0131 (8)	0.0093 (8)
C10	0.0907 (15)	0.0869 (15)	0.0601 (13)	0.0279 (12)	0.0244 (11)	0.0179 (11)
C11	0.130 (2)	0.1023 (19)	0.0465 (12)	0.0468 (17)	0.0173 (14)	−0.0015 (12)
C12	0.127 (2)	0.0812 (15)	0.0567 (13)	0.0293 (16)	−0.0111 (14)	−0.0197 (11)
C13	0.0832 (14)	0.0610 (11)	0.0621 (13)	0.0098 (11)	−0.0027 (10)	−0.0134 (9)
C14	0.0638 (11)	0.0617 (11)	0.0782 (14)	−0.0026 (9)	0.0154 (10)	0.0057 (10)

### Geometric parameters (Å, °)

**Table:**

Cl1—C4	1.742 (2)	C8—C9	1.396 (3)
O1—C7	1.2299 (19)	C9—C10	1.390 (3)
N1—C7	1.346 (2)	C9—C14	1.498 (3)
N1—C8	1.426 (2)	C10—C11	1.366 (3)
N1—H1	0.86 (2)	C10—H10	0.9400
C1—C2	1.382 (3)	C11—C12	1.363 (4)
C1—C6	1.389 (2)	C11—H11	0.9400
C1—C7	1.493 (2)	C12—C13	1.387 (3)
C2—C3	1.381 (3)	C12—H12	0.9400
C2—H2	0.9400	C13—H13	0.9400
C3—C4	1.376 (3)	C14—H14A	0.9700
C3—H3	0.9400	C14—H14B	0.9700
C4—C5	1.374 (3)	C14—H14C	0.9700
C5—C6	1.384 (3)	C14—H14D	0.9699
C5—H5	0.9400	C14—H14E	0.9700
C6—H6	0.9400	C14—H14F	0.9701
C8—C13	1.388 (3)		
			
C7—N1—C8	125.15 (15)	C9—C10—H10	118.8
C7—N1—H1	116.5 (14)	C12—C11—C10	119.6 (2)
C8—N1—H1	118.3 (14)	C12—C11—H11	120.2
C2—C1—C6	118.81 (18)	C10—C11—H11	120.2
C2—C1—C7	118.80 (16)	C11—C12—C13	120.5 (2)
C6—C1—C7	122.26 (16)	C11—C12—H12	119.7
C3—C2—C1	121.15 (19)	C13—C12—H12	119.7
C3—C2—H2	119.4	C12—C13—C8	119.7 (2)
C1—C2—H2	119.4	C12—C13—H13	120.2
C4—C3—C2	118.77 (19)	C8—C13—H13	120.2
C4—C3—H3	120.6	C9—C14—H14A	109.5
C2—C3—H3	120.6	C9—C14—H14B	109.5
C5—C4—C3	121.57 (19)	H14A—C14—H14B	109.5
C5—C4—Cl1	118.99 (17)	C9—C14—H14C	109.5
C3—C4—Cl1	119.43 (17)	H14A—C14—H14C	109.5
C4—C5—C6	119.0 (2)	H14B—C14—H14C	109.5
C4—C5—H5	120.5	C9—C14—H14D	109.5
C6—C5—H5	120.5	H14A—C14—H14D	56.0
C5—C6—C1	120.66 (19)	H14B—C14—H14D	141.0
C5—C6—H6	119.7	H14C—C14—H14D	56.5
C1—C6—H6	119.7	C9—C14—H14E	109.5
O1—C7—N1	122.69 (17)	H14A—C14—H14E	56.5
O1—C7—C1	120.33 (15)	H14B—C14—H14E	56.0
N1—C7—C1	116.95 (14)	H14C—C14—H14E	141.1
C13—C8—C9	120.40 (18)	H14D—C14—H14E	109.5
C13—C8—N1	120.71 (18)	C9—C14—H14F	109.5
C9—C8—N1	118.89 (15)	H14A—C14—H14F	141.1
C10—C9—C8	117.52 (19)	H14B—C14—H14F	56.5
C10—C9—C14	120.6 (2)	H14C—C14—H14F	56.0
C8—C9—C14	121.86 (17)	H14D—C14—H14F	109.5
C11—C10—C9	122.3 (2)	H14E—C14—H14F	109.5
C11—C10—H10	118.8		
			
C6—C1—C2—C3	−0.9 (3)	C6—C1—C7—N1	34.7 (2)
C7—C1—C2—C3	−176.85 (16)	C7—N1—C8—C13	−42.7 (2)
C1—C2—C3—C4	1.4 (3)	C7—N1—C8—C9	136.99 (18)
C2—C3—C4—C5	−0.8 (3)	C13—C8—C9—C10	1.4 (3)
C2—C3—C4—Cl1	−179.63 (15)	N1—C8—C9—C10	−178.29 (15)
C3—C4—C5—C6	−0.1 (3)	C13—C8—C9—C14	−179.35 (17)
Cl1—C4—C5—C6	178.64 (14)	N1—C8—C9—C14	1.0 (2)
C4—C5—C6—C1	0.6 (3)	C8—C9—C10—C11	0.5 (3)
C2—C1—C6—C5	−0.1 (3)	C14—C9—C10—C11	−178.77 (18)
C7—C1—C6—C5	175.69 (16)	C9—C10—C11—C12	−1.5 (3)
C8—N1—C7—O1	5.6 (3)	C10—C11—C12—C13	0.6 (4)
C8—N1—C7—C1	−172.29 (15)	C11—C12—C13—C8	1.3 (3)
C2—C1—C7—O1	32.5 (2)	C9—C8—C13—C12	−2.3 (3)
C6—C1—C7—O1	−143.28 (18)	N1—C8—C13—C12	177.40 (16)
C2—C1—C7—N1	−149.49 (17)		

### Hydrogen-bond geometry (Å, °)

**Table:**

*D*—H···*A*	*D*—H	H···*A*	*D*···*A*	*D*—H···*A*
N1—H1···O1^i^	0.86 (2)	2.07 (2)	2.9073 (18)	164.1 (17)

Symmetry codes: (i) *x*, *y*−1, *z*.
